# Physico-Chemical and Light-Induced Properties of Quinoline Azo-dyes Polymers

**DOI:** 10.3390/ijms21165755

**Published:** 2020-08-11

**Authors:** Dariusz Chomicki, Oksana Kharchenko, Lukasz Skowronski, Jolanta Kowalonek, Anna Kozanecka-Szmigiel, Dariusz Szmigiel, Vitalii Smokal, Oksana Krupka, Beata Derkowska-Zielinska

**Affiliations:** 1 Institute of Physics, Faculty of Physics, Astronomy and Informatics, Nicolaus Copernicus University in Torun, Grudziadzka 5, 87-100 Torun, Poland; chomicki@doktorant.umk.pl; 2 Faculty of Chemistry, Taras Shevchenko National University of Kyiv, 64/13 Volodymyrska St., 01601 Kyiv, Ukraine; oksana_kharchenko@ukr.net (O.K.); vitaliismokal@gmail.com (V.S.); oksana_krupka@yahoo.com (O.K.); 3 Institute of Mathematics and Physics, UTP University of Science and Technology, S. Kaliskiego 7, 85-796 Bydgoszcz, Poland; lukasz.skowronski@utp.edu.pl; 4 Faculty of Chemistry, Nicolaus Copernicus University in Torun, Gagarina 7, 87-100 Torun, Poland; jolak@umk.pl; 5 Faculty of Physics, Warsaw University of Technology, 75 Koszykowa Str., 00-662 Warszawa, Poland; anna.szmigiel@pw.edu.pl; 6 Sieć Badawcza Łukasiewicz-Instytut Technologii Elektronowej, Al. Lotnikow 32/46, 02-668 Warszawa, Poland; szmigiel@ite.waw.pl

**Keywords:** quinoline azo-dye polymers, optical properties, refractive index, surface relief grating, AFM, photoisomerization

## Abstract

We present investigation of optical and photochromic properties as well as of surface quality of thin films of novel methacrylic polymers with 8-hydroxyquinoline azo-dyes in side-chain. Additionally, thermal stability of polymer powders was examined and their glass transition temperature was determined. Optical properties (extinction coefficient and refractive index) were determined by spectroscopic ellipsometry (SE) combined with absorbance measurements. Photoresponsive behavior was investigated by determination of photoisomerization rates under irradiation with unpolarized 365 nm light, as well as by conduction of holographic grating inscription experiment. Thin film quality was determined by atomic force microscopy (AFM) measurements. Thermal analysis was performed by thermogravimetric (TG), derivative thermogravimetric (DTG) and differential scanning calorimetry (DSC) measurements. We found that optical properties as well as photoisomerization rates of investigated polymers are dependent on the substituent in the para position of the phenyl ring. Surface relief grating inscription was successfully generated only for materials with chromophores containing dimethylamino (N(CH_3_)_2_) and methyl (CH_3_) substituents, but all materials exhibited birefringence grating in the bulk. Surface of most thin films was very smooth, but its quality was impaired by neutral (H) as well as carboxyl (COOH) substituent. Thermal stability of copolymers with side-chain chromophores was improved compared to pure poly(methyl methacrylate) (PMMA).

## 1. Introduction

Azobenzene derivatives are a widely studied class of dyes due to their spectroscopic, photochromic and non-linear optical properties as well as photostability [1,2,3], which make them attractive for use in optical limiters, optical signal processing, optical data storage and holography [1,2,3,4,5]. Azobenzene is composed of two phenyl rings linked by an azo (-N=N-) bridge [1,3]. The nitrogen and carbon atoms in adjacent phenyl rings have sp^2^ hybridization and form extensive conjugated π-electron system [6]. Azobenzene can appear in two isomeric forms: Stable *trans* and metastable *cis* [1,3,7], and it can change its conformation due to reversible isomerization. This reaction can be induced by irradiation with light of the proper wavelength, or it can also happen spontaneously in the dark (*cis–trans* back reaction) [1]. In general, the absorption spectrum of azobenzene consists of two bands: Intense π-π* at about 320 nm and weak n-π* around 440 nm. When the molecule change its shape from *trans* to *cis* form, the π-π* band becomes weaker and shifts batochromically, while the n-π* band becomes more intense [1,3,8].

Modification of the chemical structure of azobenzene molecules influences their spectroscopic and photo-chemical properties as well as affects thermodynamic stability of the *cis* isomer [1,3]. Understanding the relationship between the structure of the molecule and its properties is important in the design of compounds suitable for specific applications [9]. Proper modification of the properties of molecules can be achieved by extending the conjugation length of the π-electron system or molecular planarity strengthening. In case of azobenzene, it is common practice to introduce the electron donating/withdrawing groups into the phenyl rings (D-π-A systems), which make them intramolecular charge transfer (ITC) chromophores [10,11,12,13]. Another way of tuning their properties is changing the different moieties in the phenyl rings, which are attached to the azo bridge [12,13].

Quinoline azobenzene derivatives are interesting compounds due to their optical, electrical, and thermal properties. Some of physico-chemical properties of different types of quinoline azo derivatives have been studied recently [14,15,16,17,18,19,20,21], however, there is still little knowledge about these materials and their light-induced behavior. Finding systems showing attractive optical response is very important for their practical use in photonics and optoelectronics.

In this study, we present optical and photochromic properties of thin films of new methacrylic polymers possessing covalently bonded 5-phenylazo-8-hydroxyquinoline moieties. We focused on the influence of different electron donating and electron withdrawing substituents in the para position of the phenyl ring on the surface quality of the films measured with atomic force microscopy (AFM), and on optical constants (i.e., extinction coefficient and refractive index) determined by spectroscopic ellipsometry (SE) together with absorbance measurements. Moreover, we investigated the photoisomerization behavior of all prepared quinoline azo-dye polymers upon irradiation with unpolarized 365 nm light, and determined the *trans*-*cis* isomerization rate. Furthermore, we demonstrate that diffraction gratings may be inscribed optically in the studied materials with efficiency dependent on their structure. For the two polymers, the so-called surface relief gratings (SRGs) frequently observed in azobenzene polymers were successfully generated. To the best of our knowledge, optical and light-induced properties of quinoline azo-derivatives polymers, consisting of quinoline structure bonded to N=N-aryl with different substituents, have not yet been described in the literature.

## 2. Results and Discussion

### 2.1. AFM Studies

**Figure 1:**
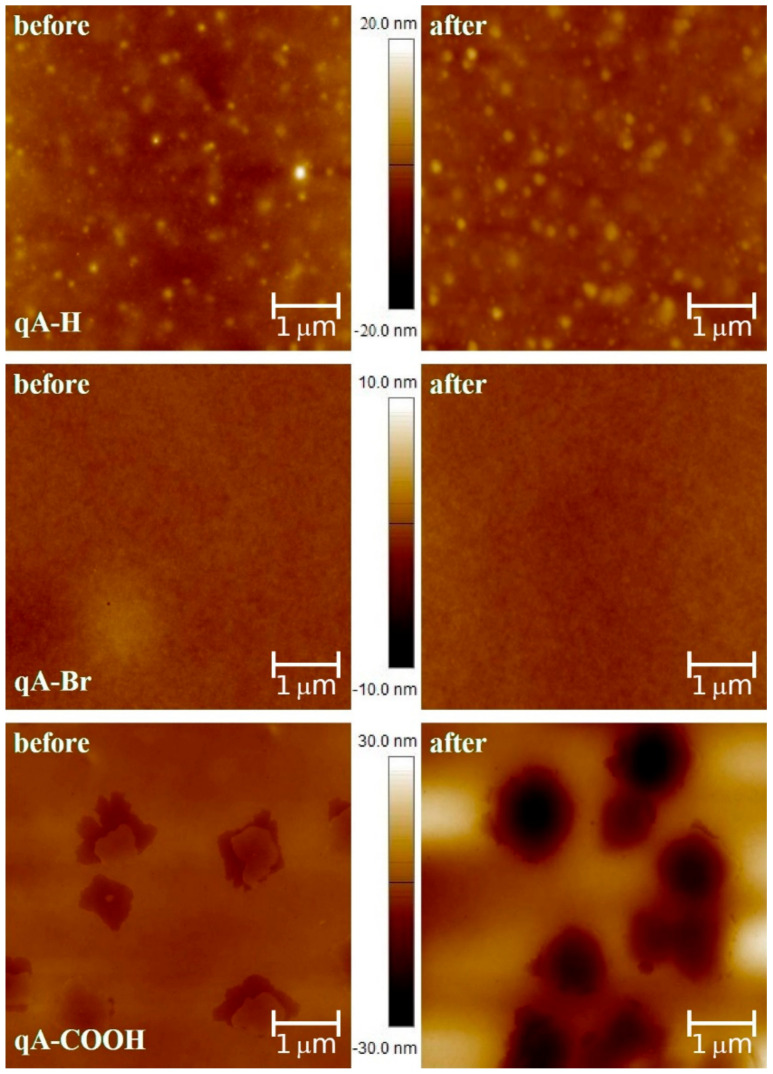
Surface topography of selected quinoline azo-polymers thin films obtained from atomic force microscopy (AFM).

AFM images for selected quinoline azo-dye containing copolymers thin films (i.e., **qA-H**, **qA-Br**, **qA-COOH**) are presented in Figure 1. The roughness parameters (*R_a_* and *R_q_*) of all studied samples were calculated to characterize their surface quality (see Table 1). One can see that most of the prepared samples had very smooth surfaces with roughness parameters values varying from 0.23 nm to 0.58 nm and from 0.31 nm to 0.86 nm for *R_a_* and *R_q_*, respectively. However, we found that the surface of **qA-H** is not so smooth (*R_a_* = 1.36 nm and *R_q_* = 1.76 nm), while **qA-COOH** had the roughest layer (*R_a_* = 1.40 nm and *R_q_* = 1.88 nm). We suppose that hydrogen bonds may have formed in **qA-COOH** before exposure, which affected morphology and roughness parameters. Afterwards, we irradiated these samples under UVA light. After 20 min, we took AFM images again and calculated their roughness parameters. We found that the visible change of the surface roughness was only observed for **qA-COOH** (*R_a_* = 7.11 nm and *R_q_* = 8.95 nm). This indicates a clear decrease in surface quality. Thus, this surface has changed. For the other samples the changes of *R_a_* and *R_q_* parameters before and after irradiation are not so large (one and a half and two times) and there is no clear tendency to these changes.

### 2.2. TGA/DSC Studies

**Figure 2:**
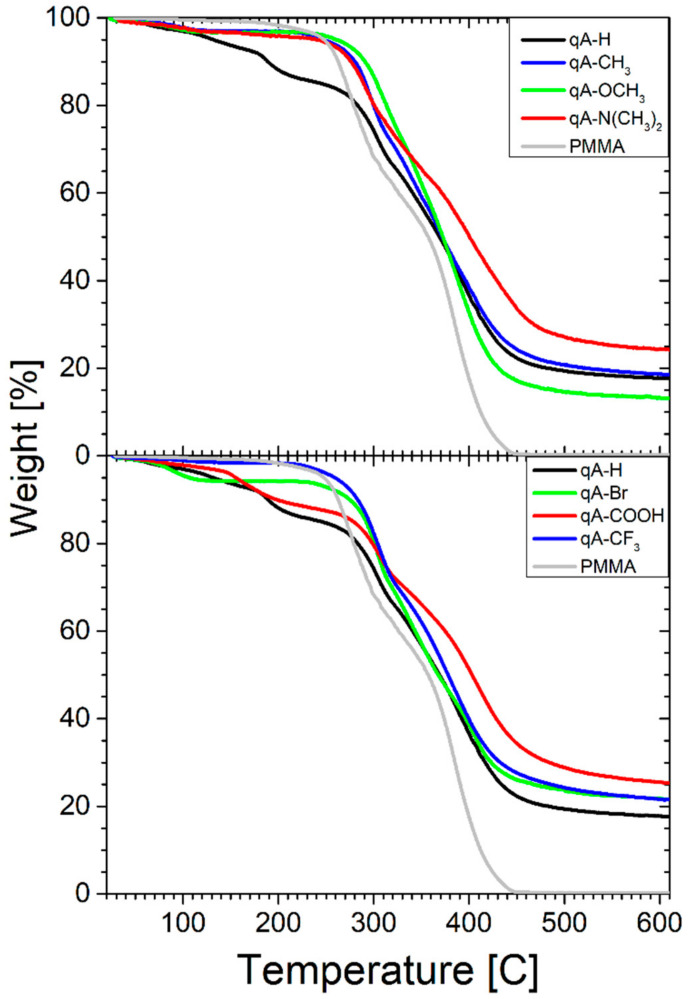
Thermogravimetric curves of PMMA and PMMA covalently bound with azo-moieties.

The thermal decomposition of poly(methyl methacrylates) (PMMA) with covalently bound azo-compounds was investigated by thermogravimetric analysis (TGA). Thermogravimetric curves are shown in Figure 2 and the results obtained from these curves are summarized in Table 2. The decomposition of neat PMMA occurs in two stages: the first stage is associated with depolymerization, which is initiated at vinylidene end groups, the second stage is related to the random main chain scission [22]. These stages are not well-separated, they partially overlap. PMMA thermal degradation results in complete sample decomposition as indicated by the lack of the char residue at 600 °C (see Figure 2). The two-stage thermal degradation was observed for other samples too, with the exception of **qA-H** and **qA-COOH**, for which a three-stage thermal decomposition was registered (Figure 2). The step at lower temperature, 188 °C for **qA-H** and 156 °C for **qA-COOH**, could be related to the decomposition of weak head to-head linkages in PMMA [22]. Moreover, for most samples, trace amounts of solvent were detected.

Table 2 and Figure 2 show that all the studied azo-polymers had considerably higher T_1max_ (T_2max_ 304 °C for **qA-H** and 306 °C for **qA-COOH**) compared to the neat PMMA, which indicated higher thermal stability of these azo-polymers. The weight loss in this stage was about twice smaller for the polymers with azo-compounds than for homopolymer suggesting stabilizing effect of azo substituents in PMMA chain on the efficiency of the PMMA decomposition probably due to presence of the azo-units in polymethacrylate. These azo-polymers contained less unit of methyl methacrylate and decomposition was not efficient.

The T_2max_ (or T_3max_ for **qA-H** and **qA-COOH**) was slightly higher for azo-polymers and the weight loss was generally smaller (but not much) for these samples in this stage, which indicated that the presence of azo-dyes as side substituents in poly(methyl methacrylate) chain did not influence this stage of the thermal degradation of the samples clearly. These results imply that reactions of random main chain scission in the azo-polymers occurred similarly to those in the neat PMMA. However, a char residue at 600 °C was much higher in the azo-polymers than in the homopolymer, indicating thermal crosslinking in the azo-polymers. As in the first stage of the thermal decomposition, the production of the monomer in the azo-polymers was much smaller compared to the neat PMMA, these azo-compounds in the latter stages were responsible for the formation of the crosslinked structures constituting the char residue.

### 2.3. UV-VIS Measurements

**Figure 3:**
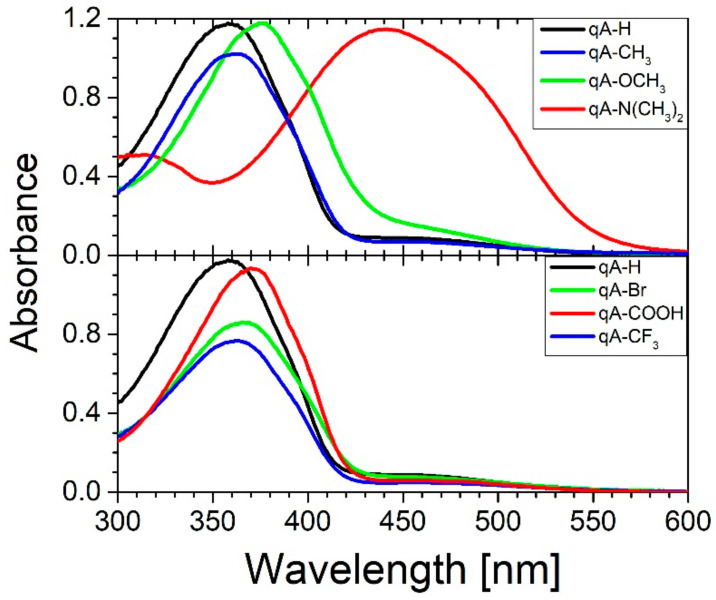
Absorbance spectra of quinoline azo-dye polymers with various electron donating and electron withdrawing substituents.

Figure 3 shows the absorbance spectra of quinoline azo-dye polymers thin films on glass substrate. All studied polymers (except **qA-N(CH_3_)_2_**) show a strong absorption band in the range of 300-420 nm resulting from π-π* electronic transition and a weak absorption band in the range of 420-550 nm assigned to n-π* transitions. The π-π* band is broad due to internal charge transfer (ITC) character of those transitions. Thus, using the classification introduced by Rau, these studied polymers might be classified to the azobenzenes category as their absorption spectra are similar to that of unsubstituted azobenzene (i.e., the absorption spectra are characterized by relatively poor overlap between the π-π* and n-π* bands) [1,2,23]. In the case of **qA-N(CH_3_)_2_**, the π-π* absorption band lies in the range of 350-550 nm and completely overlaps with the n-π* transition band. Therefore, it can be classified as pseudostilbenes type of azobenzene derivative in Rau’s classification due to total overlap of the π-π* and n-π* absorption bands and an extremely fast back conversion *cis–trans* [1,2,24].

From Figure 3 one can see that the maximum of absorption band for the neutral sample (i.e., sample without substituents-**qA-H**) is located at about 358 nm. Electron donating and electron withdrawing substituents at R position of azo-quinoline polymer thin films cause the bathochromic shift of the π-π* absorption band compared to **qA-H**. We found that this red-shift increases with an increase of electron donating strength of the substituent as well as with an increase of electron withdrawing strength of the substituent. However, in the case of **qA-CF_3_**, the bathochromic shift is the weakest although trifluoromethyl is the strongest acceptor among electron withdrawing substituents used.

### 2.4. Ellipsometric Studies

**Figure 4:**
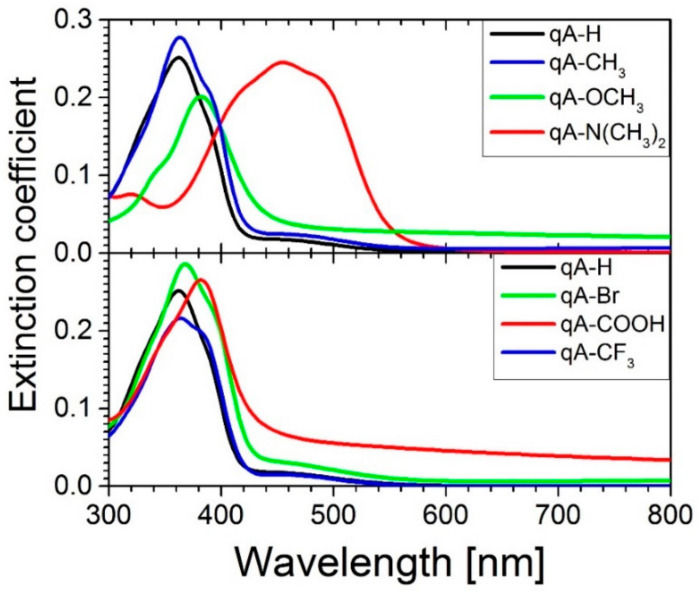
The extinction coefficient versus wavelength for quinoline azo-dye polymers with various electron donating and electron withdrawing substituents.

Figure 4 shows extinction coefficient (*k*) of the studied quinoline azo-dyes side-chain polymers obtained from spectroscopic ellipsometry (SE) measurements. It should be noted that the results obtained from SE measurements behave the same as in the case of absorption measurements. However, the energies of the interband transitions are more clearly visible in these spectra. These energies are summarized in Table 3. We used the Gaussian oscillators to parameterized the shape of extinction coefficient spectra.

**Figure 5:**
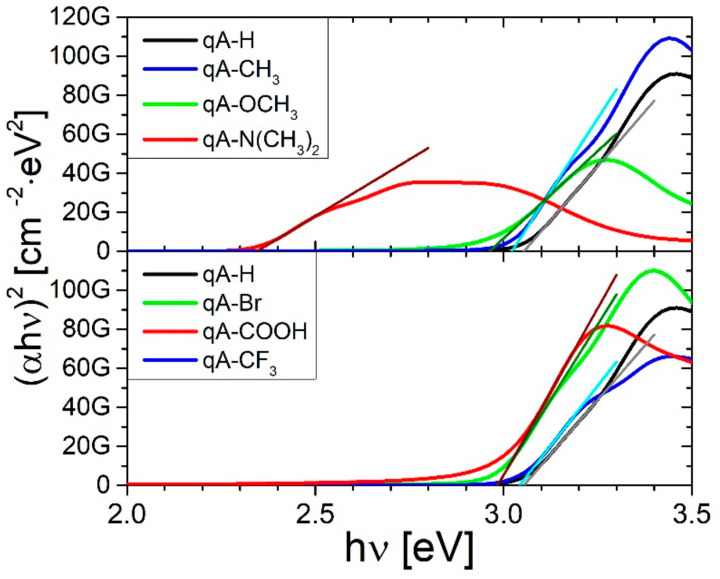
The Tauc plots of the quinoline azo-dye polymers with various electron donating and electron withdrawing substituents.

Figure 5 presents the Tauc plots of the studied azo-compounds with the extrapolation of the linear part of the (*αhν*)^2^ relation plotted as a function of photon energy. We used the Tauc method to determine the optical energy band gap (*E_g_*) for quinoline azo-dye polymers with various electron donating and electron withdrawing substituents. The values of optical energy band gap (*E_g_*) are shown in Table 3. The values of energy band gap decrease with increasing of electron donating strength, whereas the opposite behavior is visible for the polymers with electron withdrawing substituents.

**Figure 6:**
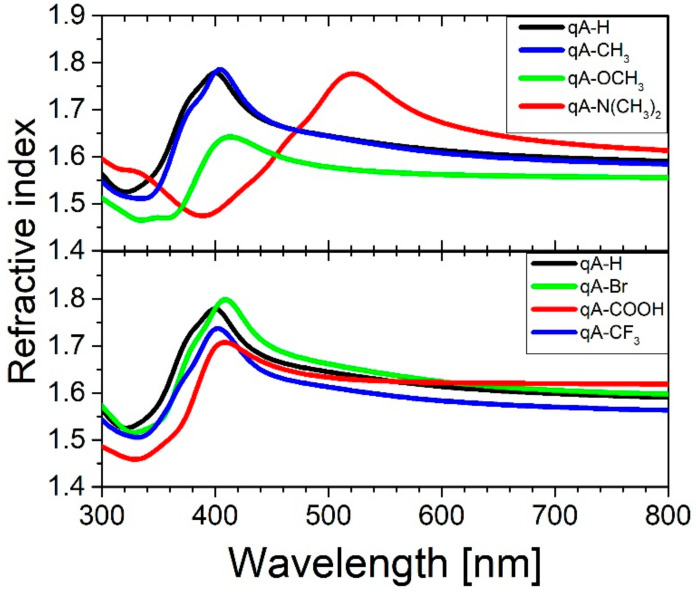
The refractive index (*n*) versus wavelength for the quinoline azo-dye polymers with various electron donating and electron withdrawing substituents.

The refractive index (*n*) of the quinoline azo-dye polymers is shown in Figure 6. In the spectral range of 300–410 nm, refractive index exhibits anomalous dispersion and in the wavelengths above 410 nm it shows normal dispersion with exception of **qA-N(CH_3_)_2_**. The substituent in the chromophore effects the position of the maximum refractive index (i.e., point of transition from anomalous to normal dispersion) in the same way as absorption maxima and this tendency was already described above. In the non-absorbing range (i.e., for wavelengths above 600 nm) the refractive index values increase in the following order *n***_qA-OCH3_** < *n***_qA-CH3_** < *n***_qA-H_** < *n***_qA-N(CH3)2_** for the polymers with electron donating substituents and in order *n***_qA-CF3_** < *n***_qA-H_** < *n***_qA-Br_** < *n***_qA-COOH_** for the polymers with electron withdrawing substituents. One can see that the refractive index value decreases as the electron donating strength increases, except **qA-N(CH_3_)_2_**. For materials with electron withdrawing substituents the tendency is reversed. It means that the *n* decreases with decreasing of the electron withdrawing strength, except **qA-CF_3_**.

### 2.5. Photoisomerization Studies

**Figure 7:**
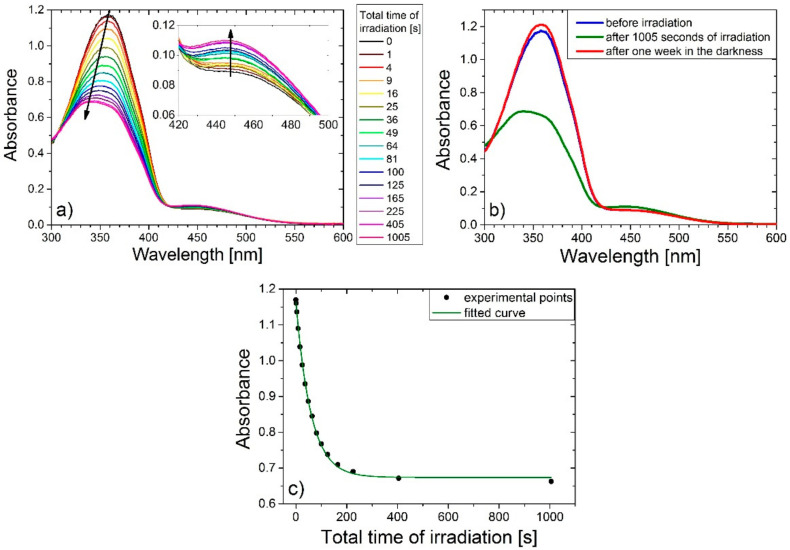
(**a**) Changes in absorption spectrum due to photoisomerization for **qA-H** sample. (**b**) Changes in absorption spectrum before and after irradiation for **qA-H**. (**c**) The evolution of the π-π* absorption band of **qA-H** as a function of the irradiation time.

Figure 7a presents the example of a decrease in the intensity of the absorption spectrum of **qA-H** during irradiation with 365 nm light. This photoisomerization behavior was found for all studied samples, except **qA-N(CH_3_)_2_**. Irradiation of the sample reduces the π-π* absorption band with simultaneous blue shift. At the same time the intensity of the n-π* band increases. The isosbestic points in the obtained spectra indicate that photoisomerization was the only reaction occurring in the samples during irradiation. We found that after *trans*-*cis* conversion resulting from UVA irradiation, the π-π* absorption band decreases and hypsochromic shifts, while the n-π* transition increases. It should be mentioned that monitoring changes in the intensity and location of absorption bands (resulting from light irradiation) can provide information on the kinetics of *trans*–*cis* and *cis*–*trans* isomerization. Then, all samples were put into darkness for one week to check if the changes in absorption spectrum were caused by photoisomerization after the irradiation. Afterwards, the absorption of azo-quinoline polymers thin films was again measured. Figure 7b shows the obtained result for **qA-H**. As you can see the spectrum returned to the shape, which it had before the irradiation of the sample, as a result of back reaction after one week in the dark [3,25]. We suppose that a slight increase of the π-π* absorption band after one week in the dark in comparison to the absorption band before irradiation can be attributed to the different orientation of chromophores after reversible *cis–trans* isomerization process compare with the initial orientation of azo- moieties in thin films. The influence of direction of azo-moieties on the absorption spectrum was showed by Kajzar [26]. Whereas, Figure 7c illustrates the evolution of the π-π* absorption band of **qA-H** as a function of the irradiation time. The exponential decay of experimental data is visible. Table 4 presents the values of the decay times and isomerization rates for all studied samples. It should be mentioned that decay time was obtained from fitting the theoretical curve (Eq. 1) to experimental data and the isomerization rate was calculated based on it. One can see that for polymers with electron donating substituents the isomerization rate increases with increasing the electron donating strength of the attached group. The changes in the absorption spectrum of **qA-N(CH_3_)_2_** upon irradiation process at λ = 365 nm were not observed, which can be explained by fast thermal back isomerization (milliseconds) at room temperature [27,28]. For the samples with electron withdrawing substituents, we can also observe an increase of the isomerization rate with increasing electron withdrawing strength, except **qA-CF_3_**. The obtained results are in good agreement with Rau’s classification for azobenzene and pseudostilbene type molecules [1,2]. Airinei et al. measured the absorption spectra of azobismaleimides ABM 1 and ABM 2 under 365 nm light irradiation. For ABM 2 in DMF solution, they obtained the photoisomerization rate constant of (3.31 ± 0.03) × 10^−2^·s^−1^. Whereas, the rate constant of photoisomerization process for ABM 1 was about two times lower than that of ABM 2 [29]. These values are similar to our results; however, it should be noted that in the case of studied quinoline azo-dye polymers the photoisomerization rate constant was measured for materials in the form of thin films.

### 2.6. Holographic Grating Inscription

The experiments on grating inscription were performed for the interfering beams with opposite circular polarization, which is the polarization combination recognized by Viswanathan et al. as the most efficient for generating the deepest surface reliefs in an azobenzene-functionalized polymer [30]. It may be assumed that in the case of the arranged recording geometry (*θ =* 7°), the resulting polarization of the optical field was linear in a direction that spatially varied across the interference region (i.e., along the transverse coordinate) [30]. As it can be seen from Figure 3, for the majority of the studied polymers, the 442 nm irradiation excited the n-π* electronic transitions, while both types of transitions (i.e., n-π* and π-π*) were excited in the case of **qA-N(CH_3_)_2_** and **qA-OCH_3_** polymers. Considering the extinction coefficients (Figure 4), one can notice that 442 nm light was much more strongly absorbed by **qA-N(CH_3_)_2_** compared to other materials.

**Figure 8:**
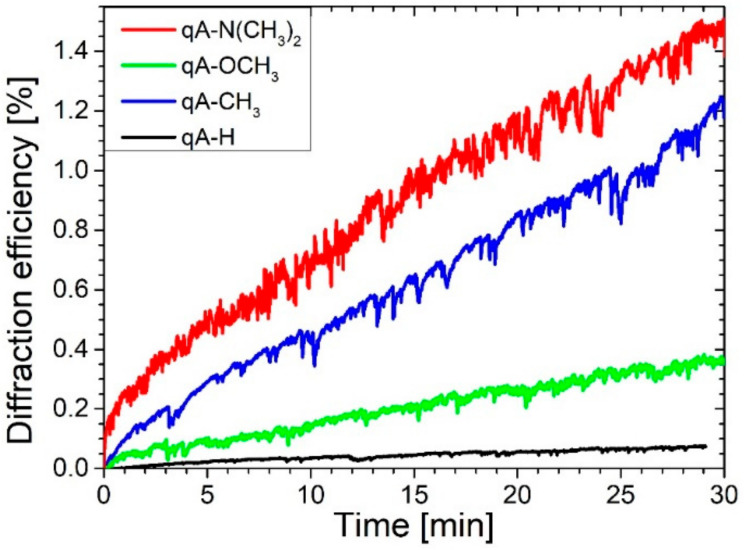
The first order diffraction efficiency as a function of time for the quinoline azo-dye polymers with various electron donating substituents. (Note that the diffraction efficiency is calculated as the ratio of the intensity of the first order diffracted beam to the incident probe beam intensity: η = I + 1/Iinc.).

As soon as the illumination started, the first order diffracted probe beam was observed for all the studied polymers proving *trans*–*cis* isomerization reactions initiated by blue excitation, and subsequent molecule reorientation processes resulting in diffraction grating formation. The intensities of the detected diffracted beams increased monotonously during 30 min of exposure as shown for the **qA-N(CH_3_)_2_** and **qA-CH_3_** samples in Figure 8.

The largest final first order diffraction efficiency (i.e., calculated as the ratio of the intensity of the first order diffracted beam at the end of illumination to the incident probe beam intensity) was ca. 1.51 % and 1.25 % for **qA-N(CH_3_)_2_** and **qA-CH_3_**, respectively. The lowest diffraction efficiency of 0.08 % exhibited **qA-H**, while the other materials diffracted the 690 nm beam with a final efficiency in the range (0.35–0.45)%. Although the diffraction efficiency of order of 0.4% is rather low, the lack of saturation of all the diffracted signals indicated a potential for achieving a more efficient diffraction in the studied series.

**Figure 9:**
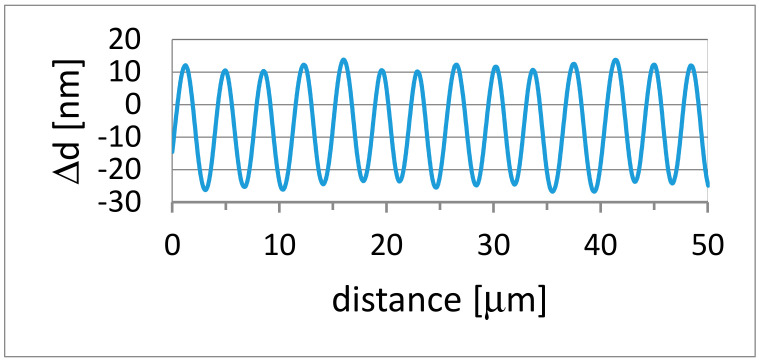
Surface profile measured for **qA-N(CH_3_)_2_** with a stylus profiler.

Examination of surface topography of all samples after illumination showed that regular relief structures with periods of 3.5 μm thus, equal to the period of the interference pattern were inscribed in **qA-N(CH_3_)_2_** and **qA-CH_3_**. The measured surface modulation profile having 37 nm-depth in case of **qA-N(CH_3_)_2_** is presented in Figure 9. The SRG amplitude found for **qA-CH_3_** was 13 nm.

The lack of relief structures on the surfaces of **qA-OCH_3_**, **qA-H**, **qA-Br** and **qA-CF_3_** explains the low diffraction efficiencies recorded for these samples, as the diffracted beams were generated by bulk birefringence gratings only. It should be noted that the highest amplitude of SRG found for **qA-N(CH_3_)_2_** with strongest electron donating substituent. It is widely known that efficiency of SRG formation in azobenzene polymers strongly depends on the irradiation conditions such as, polarization configuration of interfering beams as well as on their intersection angle. Therefore, it is reasonable to suspect that by modification of particularly the latter parameter it may be possible to observe deeper SRGs in the studied quinoline azo-polymers.

## 3. Materials and Methods

### 3.1. Materials and Synthesis

Quinolin-8-ol, N,N’-dimethyl-p-phenylenediamine (a), 4-methoxyaniline (b), 4-methylaniline (c), aniline (d), 4-bromoaniline (e), 4-aminobenzoic acid (f), and 4-(trifluoromethyl)aniline (g) were purchased from Sigma-Aldrich and used without further purification.

General procedure for synthesis of 5-azo-8-hydroxyquinoline dyes is known [11,12,20,31]. Aromatic amine (a–g) (35 mmol) was dissolved in concentrated HCl (30 mL). Some amines had to be heated before solving, and then they were kept at 0–5 °C. Sodium nitrite (35 mmol) was dissolved in 30 mL of water. Then solution of sodium nitrite was added dropwise to the previously prepared hydrochloride salts. Diazonium salt was stirring during 1 h in ice bath. An appropriate amount of quinolin-8-ol (35 mmol) was dissolved in 200 mL of ethanol and 50 mL of 10% NaOH (aq). Solution was kept at 0–5 °C during stirring. Then the solution of diazonium salt was added dropwise slowly to quinolin-8-ol containing mixture. Stirring was continued up to 1 h and pH was controlled (10–11) by the simultaneous addition of 5% sodium hydroxide aqueous solution. At the end of procedure, the pH of reaction mixture was regulated at 4–5. Then, the product was precipitated out from the solution and was kept at Buchner funnel. The crude product was purified by crystallization from EtOH or CH_3_CN:CH_3_OC_4_O_9_ to yield precipitates as follows:

*5-{(E)-[4-(dimethylamino)phenyl]diazenyl}quinolin-8-ol* (1a). Yield 55%. Mp = 212–214 °C. ^1^H NMR (DMSO-d_6_; 400 MHz) δ, ppm: 3.06 (s, 6H, CH_3_), 6.83 (d, 2H, ArH), 7.33 (d, 1H, ArH), 7.87–7.93 (m, 4H, ArH), 9.02 (d, 1H, ArH), 9.49 (d, 1H, ArH).

*5-[(E)-(4-methoxyphenyl)diazenyl]quinolin-8-ol* (1b). Yield 81%. Mp = 178–180 °C. ^1^H NMR (DMSO-d_6_; 400 MHz) δ, ppm: 3.85 (s, 3H, OCH_3_), 7.10 (d, 2H, ArH), 7.46 (d, 1H, ArH), 7.92–7.98 (m, 4H, ArH), 9.06 (d, 1H, ArH), 9.54 (d, 1H, ArH).

*5-[(E)-(4-methylphenyl)diazenyl]quinolin-8-ol* (1c). Yield 79%. Mp = 188–189 °C. ^1^H NMR (DMSO-d_6_; 400 MHz) δ, ppm: 2.39 (s, 3H, CH_3_), 7.37 (d, 2H, ArH), 7.41 (d, 1H, ArH), 7.87 (d, 2H, ArH), 7.93 (t, 1H, ArH), 8.00 (d, 1H, ArH), 9.06 (d, 1H, ArH), 9.54 (d, 1H, ArH).

*5-[(E)-phenyldiazenyl]quinolin-8-ol (1d).* Yield 90%. Mp = 174–177 °C. ^1^H NMR (DMSO-d_6_; 400 MHz) δ, ppm: 7.11 (d, 1H, ArH), 7.48 (d, 1H, ArH), 7.57 (d, 2H, ArH), 7.70 (t, 1H, ArH), 7.93 (d, 2H, ArH), 8.00 (d, 1H, ArH), 8.91 (d, 1H, ArH), 9.26 (d, 1H, ArH).

*5-[(E)-(4-bromophenyl)diazenyl]quinolin-8-ol* (1e). Yield 80%. Mp = 223–224 °C. ^1^H NMR (DMSO-d_6_; 400 MHz) δ, ppm: 7.28 (d, 1H, ArH), 7.77 (d, 2H, ArH), 7.89 (d, 2H, ArH), 8.00 (d, 1H, ArH), 8.98 (d, 1H, ArH), 9.29 (d, 1H, ArH).

*4-[(E)-(8-hydroxyquinolin-5-yl)diazenyl]benzoic acid* (1f). Yield 64%. Mp = 295–297 °C. ^1^H NMR (DMSO-d_6_; 400 MHz) δ, ppm: 6.47 (d, 1H, ArH), 7.47 (dd, 1H, ArH), 7.63 (d, 2H, ArH), 7.94 (d, 2H, ArH), 8.02 (d, 1H, ArH), 9.14 (d, 1H, ArH).

*5-{(E)-[4-(trifluoromethyl)phenyl]diazenyl}quinolin-8-ol* (1g). Yield 74%. Mp = 185 °C. ^1^H NMR (DMSO-d_6_; 400 MHz) δ, ppm: 7.26 (d, 1H, ArH), 7.77 (dd, 1H, ArH), 7.94 (d, 2H, ArH), 8.06 (d, 1H, ArH), 8.13 (d, 2H, ArH), 8.99 (d, 1H, ArH), 9.30 (d, 1H, ArH).

General procedure of synthesis of novel 8-methacryloxyquinoline azo dyes [32]. Derivatives of azo-dyes based on quinolin-8-ol (1a–1g) (3.2 mmol) were dissolved in 50 mL of acetone or tetrahydrofuran (THF) and Et_3_N (4.8 mmol) was added to stirring solution. Mixture was kept in ice bath at 0–5 °C. Then methacryloyl chloride (3.2 mmol) was added dropwise. The reaction mixture was left for stirring during 4–5 h. TLC was used for monitoring progress of acylation. Then solution was poured into ice-cool water and product was collected by filtration, then washed with a large amount of water. The target products were obtained by recrystallization from hexane or toluene: hexane as follows:

*5-{(E)-[4-(dimethylamino)phenyl]diazenyl}quinolin-8-yl-2-methylprop-2-enoate* (m1a). Brown-red powder, yield 35%. Mp = 135 °C. ^1^H NMR (DMSO-d_6_; 400 MHz) δ, ppm: 2.08 (s, 3H, CH_3_), 3.08 (s, 6H, CH_3_), 5.98 (s, 1H, CH_2_=), 6.41 (s, 1H, CH_2_=), 6.86 (d, 2H, ArH), 7.66 (d, 1H, ArH), 7.73 (t, 1H, ArH), 7.85 (d, 1H, ArH), 7.94 (d, 2H, ArH), 8.98 (d, 1H, ArH), 9.26 (d, 1H, ArH).

^13^C NMR (75 MHz, CDCl_3_) δ: 166.2, 152.8, 150.8, 148.5, 146.2, 144.4, 141.4, 135.8, 132.8, 128.5, 127.9, 127.7, 125.6, 121.8, 111.8, 111.6, 40.4, 18.7.

HRMS (MALDI-TOF DIT+) [M+H]^+^ *m*/*z*: calcd for C_21_H_21_N_4_O_2_: 361.16590; found: 361.1665.

*5-[(E)-(4-methoxyphenyl)diazenyl]quinolin-8-yl-2-methylprop-2-enoate* (m1b). Orange powder, yield 73%. Mp = 152–153 °C. ^1^H NMR (DMSO-d_6_; 400 MHz) δ, ppm: 2.10 (s, 3H, CH_3_), 3.91 (s, 3H, OCH_3_), 5.99 (s, 1H, CH_2_=), 6.43 (s, 1H, CH_2_=), 7.18 (d, 2H, ArH), 7.72 (m, 2H, ArH), 7.91 (d, 1H, ArH), 8.07 (d, 2H, ArH), 9.02 (d, 1H, ArH), 9.29 (d, 1H, ArH).

^13^C NMR (75 MHz, CDCl_3_) δ: 166.1, 162.6, 151.0, 149.5, 147.6, 145.6, 141.3, 135.7, 132.8, 128.1, 127.9, 125.4, 122.2, 121.7, 114.5, 112.3, 55.8, 18.7.

HRMS (MALDI-TOF-DIT+) [M+H]^+^ *m*/*z*: calcd for C_20_H_18_N_3_O_3_: 348.13427; found: 348.1346.

*5-[(E)-(4-methylphenyl)diazenyl]quinolin-8-yl-2-methylprop-2-enoate* (m1c). Bright orange crystals, yield 78%. Mp = 125–126 °C. ^1^H NMR (DMSO-d_6_; 400 MHz) δ, ppm: 2.09 (s, 3H, CH_3_), 2.43 (s, 3H, CH_3_), 6.00 (s, 1H, CH_2_=), 6.43 (s, 1H, CH_2_=), 7.44 (d, 2H, ArH), 7.74 (m, 2H, ArH), 7.92 (d, 1H, ArH), 7.98 (d, 2H, ArH), 9.03 (d, 1H, ArH), 9.30 (d, 1H, ArH).

^13^C NMR (75 MHz, CDCl_3_) δ 166.1, 151.3, 151.1, 150.0, 145.5, 142.3, 141.4, 135.7, 132.6, 130.0, 128.1, 128.1, 123.4, 122.3, 121.5, 112.4, 21.7, 18.7.

HRMS (MALDI-TOF-DIT+) [M+H]^+^ *m*/*z*: calcd for C_20_H_18_N_3_O_2_: 332.13935; found: 332.1396.

*5-[(E)-phenyldiazenyl]quinolin-8-yl-2-methylprop-2-enoate* (m1d). Orange powder, yield 51%. Mp = 128–129 °C. ^1^H NMR (DMSO-d_6_; 400 MHz) δ, ppm: 2.09 (s, 3H, CH_3_), 6.01 (s, 1H, CH_2_=), 6.43 (s, 1H, CH_2_=), 7.64 (m, 3H, ArH), 7.75 (m, 2H, ArH), 7.95 (d, 1H, ArH), 8.07 (d, 2H, ArH), 9.04 (d, 1H, ArH), 9.31 (d, 1H, ArH).

^13^C NMR (75 MHz, CDCl_3_) δ 166.1, 153.1, 151.1, 150.3, 145.4, 141.4, 135.7, 132.6, 131.6, 129.4, 128.2, 123.4, 123.0, 122.4, 121.6, 112.6, 18.7.

HRMS (MALDI-TOF-DIT+) [M+H]^+^ *m*/*z*: calcd for C_19_H_16_N_3_O_2_: 318.12370; found: 318.1238.

*5-[(E)-(4-bromophenyl)diazenyl]quinolin-8-yl-2-methylprop-2-enoate* (m1e). Bright orange crystals, yield 64%. Mp = 141–142 °C. ^1^H NMR (DMSO-d_6_; 400 MHz) δ, ppm: 2.09 (s, 3H, CH_3_), 6.01 (s, 1H, CH_2_=), 6.43 (s, 1H, CH_2_=), 7.75 (d, 1H, ArH), 7.79 (d, 1H, ArH), 7.84 (d, 2H, ArH), 7.96 (d, 1H, ArH), 8.01 (d, 2H, ArH), 9.03 (d, 1H, ArH), 9.30 (d, 1H, ArH).

^13^C NMR (75 MHz, CDCl3) δ 166.0, 151.8, 151.2, 150.6, 145.2, 141.4, 135.6, 132.6, 132.4, 128.24, 128.18, 126.1, 124.8, 122.5, 121.6, 112.7, 18.7

HRMS (MALDI-TOF-DIT+) [M+H]^+^ *m*/*z*: calcd for C_19_H_15_N_3_O_2_Br: 396.03422; found: 396.0346.

*4-[(E)-{8-[(2-methacryloyl)oxy]quinolin-5-yl}diazenyl]benzoic acid* (m1f). Brick red powder, yield 40%. Mp = 260–265 °C. ^1^H NMR (DMSO-d_6_; 400 MHz) δ, ppm: 2.09 (s, 3H, CH_3_), 6.01 (s, 1H, CH_2_=), 6.43 (s, 1H, CH_2_=), 7.78 (d, 2H, ArH), 7.99 (d, 1H, ArH), 8.16 (d, 4H, ArH), 9.05 (d, 1H, ArH), 9.33 (d, 1H, ArH).

^13^C NMR (75 MHz, DMSO) δ 166.6, 165.1, 154.6, 151.5, 150.4, 144.5, 140.5, 134.9, 133.2, 132.0, 130.6, 128.4, 127.2, 123.3, 123.1, 121.8, 112.7, 18.1.

HRMS (MALDI-TOF-DIT+) [M+H]^+^ *m*/*z*: calcd for C_20_H_16_N_3_O_4_: 362.11353; found: 362.1129.

*5-{(E)-[4-(trifluoromethyl)phenyl]diazenyl}quinolin-8-yl-2-methylprop-2-enoate* (m1g). Bright orange crystals, yield 41%. Mp = 138–140 °C. ^1^H NMR (DMSO-d_6_; 400 MHz) δ, ppm: 2.09 (s, 3H, CH_3_), 6.02 (s, 1H, CH_2_=), 6.44 (s, 1H, CH_2_=), 7.79 (m, 2H, ArH), 8.01 (m, 3H, ArH), 8.25 (d, 2H, ArH), 9.05 (d, 1H, ArH), 9.33 (d, 1H, ArH).

^13^C NMR (75 MHz, CDCl_3_) δ 166.0, 154.7, 151.2, 151.0, 145.1, 141.3, 135.6, 133.0, 132.6, 132.5, 128.4, 126.6, 126.5, 123.5, 122.7, 121.6, 113.0, 18.7.

HRMS (MALDI-TOF-DIT+) [M+H]^+^ *m*/*z*: calcd for C_20_H_15_N_3_O_2_F_3_: 386.11109; found: 386.1108.

#### 3.1.1. Polymerization

Azo-quinoline containing methacrylic copolymers were synthesized by free radical polymerization in 10 % N,N’-dimethylformamide (DMF) [33,34]. The methylmethacrylate (MMA) (1.8 mmol) and m1a–m1g (0.6 mmol) and 2,2′-azobisisobutyronitrile (AIBN) (1 wt %) were dissolved in degassed anhydrous DMF. The reaction mixture was kept at 80 °C for 24 h.

The solution was poured into methanol to precipitate the copolymer product. The dissolution precipitation cycle was repeated twice using THF solution and methanol to further purify copolymer. The comonomer ratio in polymers was determined by the integration of ^1^H NMR signals.

**Figure 10:**
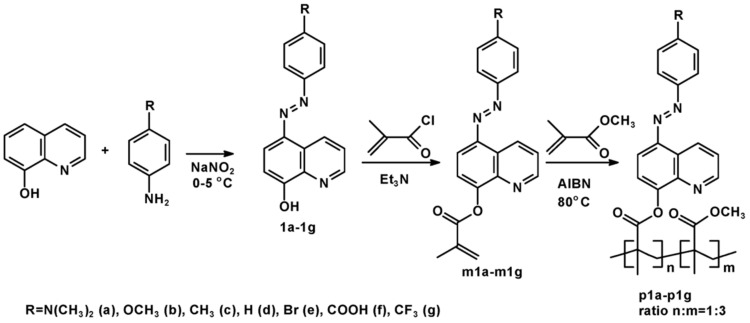
Synthesis of azo-quinoline containing polymers with various electron donating and electron withdrawing substituents.

The synthetic route of studied methacrylic copolymers containing 8-hydroxy-quinoline azo-dyes in the side chain is presented in Figure 10. As was mentioned above, the copolymers are composed of methylmethacrylate unit and methacrylic azoesters, in which chromophore is covalently bonded as side chain. The copolymerization ratio is in reasonable accordance with initial amounts of respective monomers (3:1), respectively.

Chromophores are composed of 8-hydroxy-quinoline moiety and phenyl ring linked through an azo bridge. Various substituents were attached at the para position of the phenyl ring (R) with different electron donating/withdrawing strength. The Hammett substituent constants for corresponding samples are presented in Table 5. It is obvious that the **qA-N(CH_3_)_2_** polymer has the strongest electron donating substituent (i.e., dimethylamino group, -N(CH_3_)_2_) in para position, whereas **qA-CF_3_** polymer has the strongest electron-withdrawing substituent (i.e., trifluoromethyl, CF_3_ group). The neutral substituent is present in **qA-H**.

#### 3.1.2. Density Functional Theory Geometry Optimization

Geometry of the chromophores was optimized with density functional theory (DFT) calculations. These calculations were performed using BP86 exchange-correlation functional and DZP basis set. It should be noted that they were made for the isolated molecule in a vacuum. The magnitudes of molecule’s dipole moment were determined and they are listed in Table 5. One can see that value of molecule’s dipole moment increases with increasing of the electron donating/withdrawing strength.

### 3.2. Sample Preparation

Thin films of copolymers with quinoline azo-dye moiety were deposited by spin-coating method. Droplets of 100 g/L solutions of polymers in 1,2-dichloroethane were casted onto immobile substrates and then spun. It should be mentioned that thin films were deposited on glass substrates for transmission, photoisomerization and AFM studies. Whereas, thin layers formed on silicon substrates were used for ellipsometric measurements.

Thin films for AFM studies were deposited using Microprocessor-Controlled Spin Coater SCC-200 (Novocontrol Technologies GmbH & Co. KG, Montabaur, Germany). Spin-coating procedure was divided in two parts. After dropping the polymer solution onto the substrate, this substrate was initially accelerated (with acceleration 480 rpm/s) until their speed reached 2400 rpm. Then, after 30 s, it was again accelerated (with acceleration 240 rpm/s) to speed of 3600 rpm, and after 30 s it was stopped. Whereas, thin layers for all other measurements were formed with Compact Coater Cy-Ez4 (Zhengzhou CY Scientific Instrument Co., Ltd., Zhengzhou, China). The speed of spin-coating was 2000 rpm, acceleration was 1000 rpm/s and time of the whole procedure was 60 s.

### 3.3. Material Characterization

#### 3.3.1. NMR and Mass Spectra

^1^H NMR (400 MHz) spectra were recorded on a Mercury (Varian Inc., Palo Alto, California, USA) spectrometer in DMSO-d_6_ at 21 °C. Chemical shifts are given in ppm from tetramethylsilane. ^13^C NMR spectra were obtained on Bruker NMR (Bruker Corp., Karlsruhe, Germany) spectrometer (75 MHz for ^13^C-NMR). ^13^C NMR chemical shifts were reported using the solvent residual signal as an internal reference (CDCl_3_: δ_C_= 77.16 ppm; CD_3_SOCD_3_: δ_C_= 39.50 ppm). High resolution mass spectrometry (HRMS) was performed using a JEOL JMS-700 B/E machine (Jeol Ltd., Tokyo, Japan).

#### 3.3.2. Thermal Measurements

Thermal Analysis SDT 2960 Simultaneous DSC-TGA analyzer (TA Instruments, New Castle, DE, USA) was used for recording the thermal decomposition of the samples in nitrogen atmosphere and with the heating rate of 10 °C/min to 600 °C/min. Module TGA-DTA was applied. The characteristic parameters such as: T_max_ (°C)—the temperature at the maximum degradation rate (maximum on DTG curve), Δm (%)—weight loss, also char residue (%) at 600 °C were determined from thermogravimetric (TG) and derivative thermogravimetric (DTG) curves. The glass transition temperatures of copolymers with azo moiety in side chain measured by differential scanning calorimetry (DSC) are presented in Table 6.

#### 3.3.3. Atomic Force Microscope

Atomic Force Microscope (AFM) was used to examine the topology of all studied azo-polymer thin films on glass substrate. MultiMode NanoScope IIIa (Veeco Metrology Inc., Santa Barbara, CA, USA) device was used to obtain the images. The device was operating in tapping mode in air at room temperature and the standard silicon tips (Veeco) were used. Measurements were performed on as prepared samples as well as after 20 min of illumination under 365 nm UVA light with irradiance of 2.15 W/m^2^ (F6T5BLB 6W lamp from Roger Electronic Products Co., Ltd.). The imaging scan size was 5 μm × 5 μm. The roughness parameters *R_a_* ($R_{a}=\frac{1}{N}\sum_{i=1}^{N}\left|y_{i}\right|$ − arithmetical mean deviation) and *R_q_* ($R_{q}=\sqrt{\frac{1}{N}\sum_{i=1}^{N}y_{i}^{2}}$ − root mean squared deviation) were determined using NanoScope Analysis software (version 1.40) for the whole scan area.

#### 3.3.4. UV-Vis Spectra

Absorption spectra of quinoline azo-polymer thin films on glass substrates were measured using Cary 5000 (Agilent, Santa Clara, CA, USA) in the spectral range 300–600 nm.

#### 3.3.5. Ellipsometry Measurements

Whereas, extinction coefficient and refractive index as well as thickness of the azo-quinoline containing copolymers thin films on silicon substrate were determined using spectroscopic ellipsometry (V-VASE ellipsometer from J.A.Woollam Co., Inc., Lincoln, NE, USA) in the wavelength range from 248 nm to 2000 nm (5.0 eV–0.6 eV). Ellipsometric azimuths (Ψ, Δ) were measured for three angles of incidence (65°, 70°, 75°) [36]. The optical constants and thickness of studied samples were determined using the following optical model of the sample: silicon/silicon oxide/polymer layer. These optical constants were parameterized using Gauss-shape dispersion relation in the absorption regime.

#### 3.3.6. Light-Responsive Behavior Measurements

Photoisomerization studies were performed on quinoline azo-polymer thin films deposited on glass substrates. Samples were irradiated under RR-818 UV lamp (Jiady, Shenzhe, China) (4 × 9 W 365 nm bulbs). UV-VIS spectra were recorded for different times of irradiation and accumulated time of irradiation is given. Mono-exponential absorbance decay was fitted using the following formula [37]:

(1) $$A\left(t\right)=A_{PSS}+\Delta A\cdot \exp\left(-\frac{t}{\tau }\right)=A_{PSS}+\Delta A\cdot \exp\left(-pt\right)$$

where, *A_PSS_* is absorbance of material in photo-stationary state and *p* is photoisomerization rate.

#### 3.3.7. Holographic Grating Inscription

**Figure 11:**
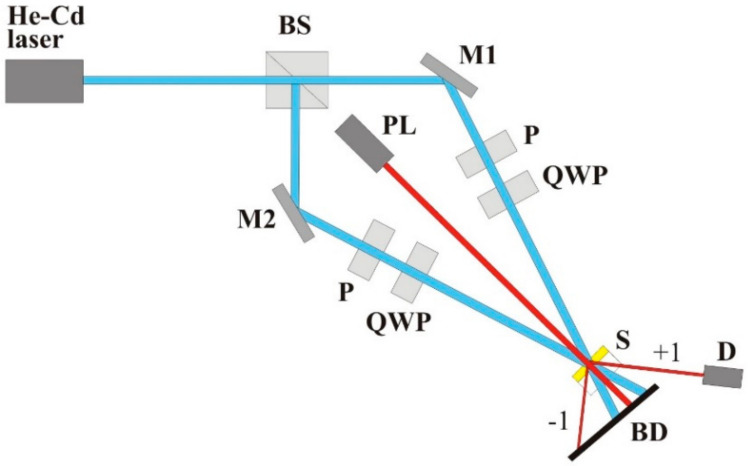
Experimental set-up for holographic grating inscription. S-sample, BS, beam splitter, M1, M2-mirrors, P-polarizer, QWP-quarter-wave plate, PL-probe laser, D- silicon detector, BD-beam dump.

The ability of all studied polymers (apart from **qA-COOH**) to generate diffraction gratings upon irradiation with light interference pattern was examined using a 442 nm He-Cd laser as a source of coherent beam. A scheme of the built experimental set-up is presented in Figure 11.

After passing through a cube beam splitter, the two output beams were guided by reflecting mirrors to cross at the angle of ca. 7°. The sample in the form of polymer layer on glass substrates was placed in the area of the beam interference. For the arranged experimental geometry, the period of light interference pattern (*Λ*) was equal to ca. 3.6 μm (according to the formula: *Λ*=λ/(2sin(*θ*/2), where *λ* is the writing wavelength, *θ* is the intersection angle [30]). The experiments were performed for the beams having intensities of 45 mW/cm^2^ and possessing opposite circular polarizations produced by properly oriented quarter-wave plates. The second 690 nm light beam was used for monitoring the process of grating formation with time. The intensity of the +1 diffraction order was measured for ca. 30 min with a silicon photodetector. After irradiation, topography of the polymer surface was examined using a Dektak XT stylus profiler.

## 4. Conclusions

A series of novel quinoline azo-polymers was synthesized and comprehensively characterized. Quality of the prepared thin films was studied with AFM measurements. The collected images showed that most of examined layers had smooth surface. We found that after irradiating the samples under UVA light, no changes were observed, except for **qA-COOH**. We suppose that hydrogen bonds may have formed in **qA-COOH** before exposure, which affected morphology and roughness parameters before and after irradiation.

Optical properties of the materials were studied with spectroscopic ellipsometry combined with absorbance measurements. The results showed that electron donating and electron withdrawing substituents in para position of phenyl ring caused bathochromic shift of absorption band. Red-shift of absorption band was higher, the higher was electron donating strength of substituent. In case of electron withdrawing substituents, the strongest electron acceptor caused the weakest red-shift of absorption band. It should be noted that the absorption band of quinoline azo-polymer with dimethylamino group with the highest electron donating strength, lied in the range of 350–550 nm and its π-π* and n-π* transition bands completely overlapped. Refractive index shifted in the same way as absorption band. In non-absorbing range, for materials with electron donating substituents the refractive index increased with the electron donating strength, except for *n***_qA-N(CH3)2_** due to the red shift of the absorption band. Whereas for quinoline azo-polymers with electron withdrawing substituents we found that the *n* changed in order *n***_qA-CF3_** < *n***_qA-H_** < *n***_qA-Br_** < *n***_qA-COOH_**. Absorbance studies on the samples irradiated with 365 nm unpolarized light revealed that thin films of investigated polymers showed photoisomerization. The changes in the absorption spectrum of **qA-N(CH_3_)_2_** upon 365 nm irradiation were not observed due to fast back isomerization. We showed that the presence of electron donating and electron withdrawing substituents resulted in an increase of the isomerization rate. However, the presence of a strong electron donating group (**qA-OCH_3_**) causes a significant increase in the *cis*–*trans* rate constant.

The experiments on holographic inscription of diffraction gratings confirmed that quinoline azo-dyes not only isomerize in polymeric system but also reorient upon irradiation with linearly polarized blue optical field, as diffracted beams were observed for all the samples. Apart from bulk birefringence gratings, surface relief structures were generated, but only in the two materials with electron donating substituents (i.e., **qA-N(CH_3_)_2_** and **qA-CH_3_**). The largest value of the first order diffraction efficiency of 1.51%, resulting predominantly from the formation of the highest-amplitude relief of 37 nm was observed for **qA-N(CH_3_)_2_**.

Our studies showed that quinoline azo-polymers thin films can be promising candidates in the field of photonics and optoelectronics. Proper design of quinoline azo-polymers can increase the rate of *cis–trans* isomerization, which can be much faster than in the case of azobenzene compounds widely studied from the point of view of their use in new photonic and optoelectronic devices.

## Figures and Tables

**Table 1:** Sample thickness (L) and values of roughness parameters (*Ra*, *Rq*) before and after irradiation.

	Irradiation	qA-N(CH_3_)_2_	qA-OCH_3_	qA-CH_3_	qA-H	qA-Br	qA-COOH	qA-CF_3_
*R_a_* (nm)	before	0.39	0.58	0.36	1.36	0.33	1.40	0.39
	after	0.24	0.28	0.44	1.46	0.37	7.11	0.25
*R_q_* (nm)	before	0.49	0.86	0.56	1.76	0.43	1.88	0.72
	after	0.34	0.38	0.53	1.96	0.50	8.95	0.54
L (nm)		672 ± 2	680 ± 5	621 ± 2	485 ± 1	499 ± 2	368 ± 3	558 ± 1

**Table 2:** Thermogravimetric parameters: temperature at maximal rate of the process (Tmax (°C)), weight loss (Δm (%)) char residue at 600 °C for pure poly(methyl methacrylate) (PMMA) and PMMA covalently bound with azo- moieties. The numbers accompanying temperatures and weight loss refer to the subsequent stages of decomposition.

Parameters	PMMA	qA-N(CH_3_)_2_	qA-OCH_3_	qA-CH_3_	qA-H	qA-Br	qA-COOH	qA-CF_3_
T_1max_ (°C)	275	292	311	296	188	302	156	305
Δ_m1_ (%)	41.5	32.6	24.0	25.4	7.0	24.6	9.4	29.0
T_2max_ (°C)	385	400	389	370	304	404	306	381
Δ_m2_ (%)	58.5	38.1	57.3	51.8	21.0	46.8	17.6	46.7
T_3max_ (°C)	-	-	-	-	387	-	409	-
Δ_m3_ (%)	-	-	-	-	46.8	-	44.3	-
Char yield (%) at 600 °C	0	24.4	13.7	18.7	17.8	21.8	25.5	21.6

**Table 3:** The values of energy band gap (*E_g_*) and energies of interband transitions for quinoline azo-dye polymers with various electron donating and electron withdrawing substituents.

Energies		qA-N(CH_3_)_2_	qA-OCH_3_	qA-CH_3_	qA-H	qA-Br	qA-COOH	qA-CF_3_
*E_g_*	(eV) (nm)	2.34 ± 0.05 530 ± 11	2.96 ± 0.05 419 ± 7	3.02 ± 0.06 411 ± 8	3.05 ± 0.05 407 ± 10	2.98 ± 0.05 416 ± 7	2.99 ± 0.05 415 ± 7	3.04 ± 0.07 408 ± 9
E_1_	(eV) (nm)	2.48 ± 0.01 499 ± 2	3.21 ± 0.03 386 ± 4	2.64 ± 0.03 470 ± 6	2.74 ± 0.02 453 ± 3	2.68 ± 0.11 463 ± 19	3.19 ± 0.04 389 ± 5	2.71 ± 0.02 458 ± 4
E_2_	(eV) (nm)	2.69 ± 0.03 460 ± 6	3.38 ± 7.74 367 ± 200	3.13 ± 0.02 396 ± 3	3.15 ± 0.01 394 ± 2	3.10 ± 0.02 400 ± 3	3.38 ± 0.03 367 ± 3	3.18 ± 0.01 390 ± 1
E_3_	(eV) (nm)	2.87 ± 0.03 432 ± 5	3.38 ± 0.04 367 ± 4	3.35 ± 0.03 370 ± 3	3.62 ± 0.03 343 ± 3	3.33 ± 0.03 372 ± 4	4.00 ± 0.27 310 ± 22	3.38 ± 0.04 367 ± 4
E_4_	(eV) (nm)	3.55 ± 0.22 349 ± 23	3.63 ± 0.12 342 ± 11	3.61 ± 0.04 343 ± 4	4.27 ± 0.03 290 ± 2	3.66 ± 0.10 339 ± 9	4.90 ± 0.12 253 ± 6	3.44 ± 0.04 360 ± 5
E_5_	(eV) (nm)	3.84 ± 0.06 323 ± 5	4.75 ± 0.05 261 ± 3			3.69 ± 0.12 336 ± 11		3.69 ± 0.04 336 ± 4
E_6_	(eV) (nm)							4.78 ± 0.09 259 ± 5

**Table 4:** Decay times, isomerization rates and isosbestic points for quinoline azo-dye polymer thin films.

	qA-N(CH_3_)_2_	qA-OCH_3_	qA-CH_3_	qA-H	qA-Br	qA-COOH	qA-CF_3_
Decay time τ (s)	-	38.7 ± 0.9	58.58 ± 0.71	58.9 ± 1.4	55.3 ± 2.6	50.2 ± 1.1	71.0 ± 3.4
Isomerization rate *p* (s^−1^)	-	(25.9 ± 0.6) × 10^−3^	(17.07 ± 0.21) × 10^−3^	(16.97 ± 0.41) × 10^−3^	(18.09 ± 0.83) × 10^−3^	(19.92 ± 0.42) × 10^−3^	(14.1 ± 0.7) × 10^−3^
Isosbestic points (nm)	-	329	310	306	311	312	303
		453	427	421	433	426	425

**Table 5:** The values of Hammett sigma constants of various substituents [35] and magnitudes of molecule’s dipole moment.

	qA-N(CH_3_)_2_	qA-OCH_3_	qA-CH_3_	qA-H	qA-Br	qA-COOH	qA-CF_3_
Types of substituents	electron donating group			neutral	electron withdrawing group		
R	N(CH_3_)_2_	OCH_3_	CH_3_	H	Br	COOH	CF_3_
R’s Hammett sigma constant	−0.83	−0.27	-0.17	0	0.23	0.45	0.54
Dipole moment (Debye)	6.5	3.0	2.3	1.7	1.9	6.2	3.6

**Table 6:** Copolymers from radical polymerization of 10% methacrylic monomers in N,N′-dimethylformamide (DMF) at 80 °C (argon atmosphere, initiator—AIBN 1%).

	qA-N(CH_3_)_2_	qA-OCH_3_	qA-CH_3_	qA-H	qA-Br	qA-COOH	qA-CF_3_
Initial monomers mole ratio n/m	1:3						
ratio n/m ^a^	1:3.1	1:3.0	1:3.2	1:3.2	1:3.1	1:3.1	1:2.9
M_w_^b^ (g/mol)	24800	36000	28100	25500	32000	25000	33200
M_w_/M_n_^b^	1.7	2.0	1.9	1.8	1.9	1.83	1.61
T_g_^c^ (°C)	-	121	122	135	94	129	128

^a^—Determined by ^1^H NMR integration; ^b^—Measured by GPC; ^c^—Measured by DSC.
